# Stabilization of α-synuclein oligomers using formaldehyde

**DOI:** 10.1371/journal.pone.0216764

**Published:** 2019-10-11

**Authors:** Harm Ruesink, Lasse Reimer, Emil Gregersen, Arne Moeller, Cristine Betzer, Poul Henning Jensen

**Affiliations:** 1 Danish Research Institute of Translational Neuroscience—DANDRITE, Aarhus University, Aarhus, Denmark; 2 Department of Biomedicine, Aarhus University, Aarhus, Denmark; 3 Department of Structural Biology, The Max Planck Institute of Biophysics, Frankfurt, Germany; National Institutes of Health, UNITED STATES

## Abstract

The group of neurodegenerative diseases, Parkinson's disease (PD), dementia with Lewy bodies (DLB), and multiple system atrophy (MSA) all exhibit inclusions containing amyloid-type α-synuclein (α-syn) aggregates within degenerating brain cells. α-syn also exists as soluble oligomeric species that are hypothesized to represent intermediates between its native and aggregated states. These oligomers are present in brain extracts from patients suffering from synucleinopathies and hold great potential as biomarkers. Although easily prepared *in vitro*, oligomers are metastable and dissociate over time, thereby complicating α-syn oligomer research. Using the small amine-reactive cross-linker, formaldehyde (FA), we successfully stabilized α-syn oligomers without affecting their size, overall structure or antigenicity towards aggregate-conformation specific α-syn antibodies FILA and MJFR-14-6-4-2. Further, cross-linked α-syn oligomers show resistance towards denaturant like urea and SDS treatment and remain fully functional as internal standard in an aggregation-specific enzyme-linked immunosorbent assay (ELISA) despite prior incubation with urea. We propose that FA cross-linked α-syn oligomers could serve as important calibrators to facilitate comparative and standardized α-syn biomarker studies going forward.

## Introduction

Parkinson’s disease (PD), Dementia with Lewy Bodies (DLB) and Multiple System Atrophy (MSA) are the three main types of synucleinopathies, all of which are characterized by inclusions mainly consisting of intracellular aggregated α-synuclein (α-syn); a 140-amino acid protein with hypothesized unfolded native appearance [1, 2]. α-syn is widely expressed in the brain and mainly localizes to the presynaptic terminals where it is believed to play a role as a SNARE-complex chaperone, necessary for binding of vesicles and the release of neurotransmitter in the synaptic cleft [3, 4]. Proof of the involvement of α-syn in PD is evident from familial cases, where specific point mutations or multiple copies of the normal α-syn gene, SNCA, causes autosomal PD [5–8]. Under normal physiological conditions, α-syn exists as a native species in the brain, but aggregates into larger insoluble fibrils under pathological conditions [9]. However, α-syn also exists in an intermediate state, as an aggregated soluble species, called oligomers. Oligomers have been found in post-mortem brain extracts from both DLB, PD and MSA patients [10–12], and accumulating evidence indicates that oligomers are responsible for the toxicity seen in the brain of patients suffering from synucleinopathies [13–15]. Yet, the exact structure of *in vivo* α-syn oligomers has yet to be determined. α-syn oligomers generated *in vitro* are capable of seeding and mediate cell-to-cell spreading linked to the prion-like spreading seen in PD [16]. α-syn oligomers can be generated in vitro in several ways, such as chemical modification by dopamine, ethanol and Fe^3+^ and high protein concentration [17–19]. Oligomers formed spontaneously at high protein concentration have shown similarities with patient-derived *in vivo* oligomers in the antigenicity towards aggregation-specific antibodies [10]. However, these oligomers are metastable and dissociate into monomers over time [20]. Therefore, an *in vivo* representative α-syn oligomer-standard used to study the underlying mechanistic pathways and aid in the search for synucleinopathy-specific biomarkers, is of great interest. Using formaldehyde (FA) cross-linking, we have successfully stabilized α-syn in its oligomeric state without disturbing antigenicity and biochemical properties, thereby providing a much-needed calibrating tool, which enables comparative and standardized research within the field of oligomeric α-syn.

## Results

### Characterizing native α-syn oligomers

**Fig 1:**
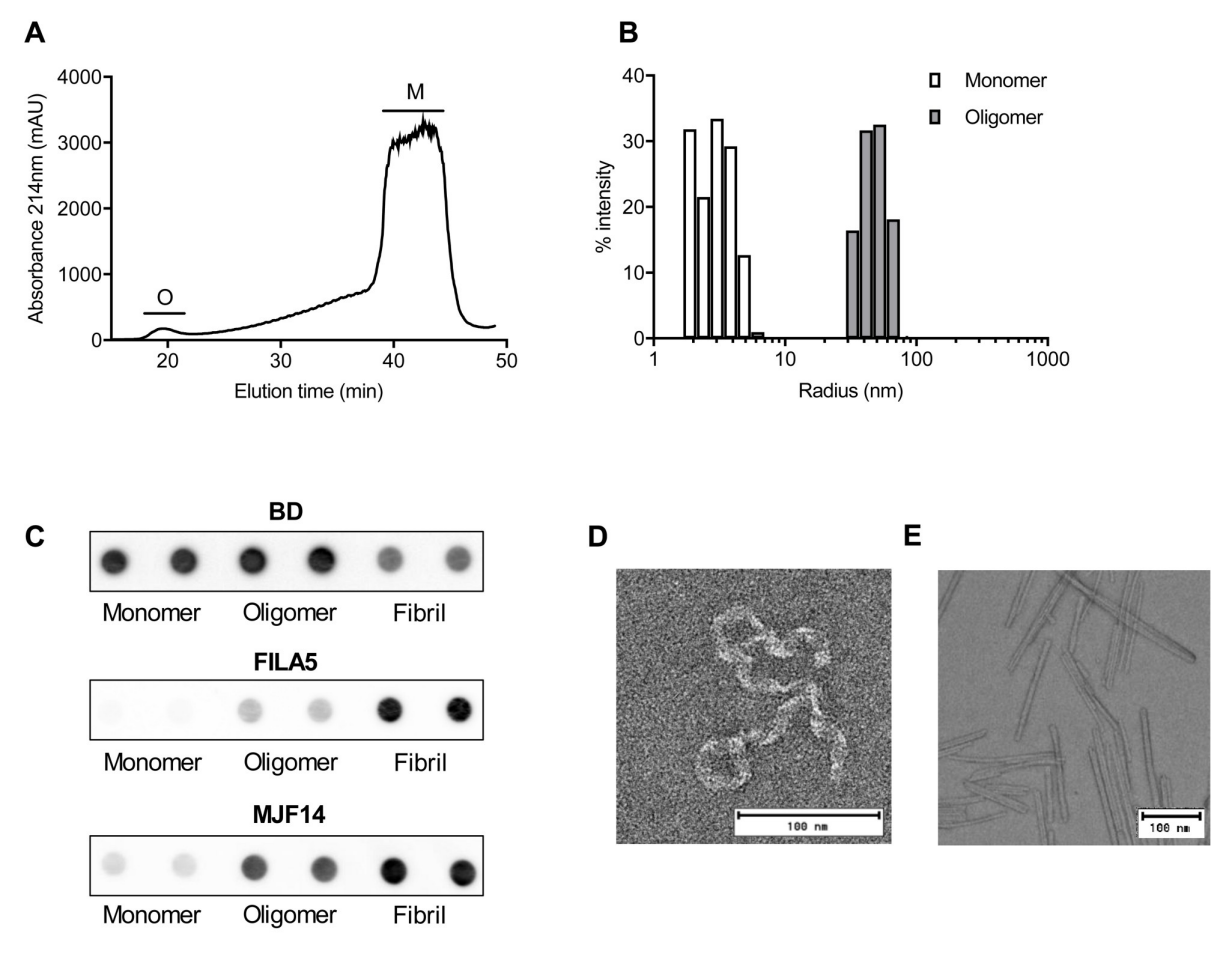
Generation and confirmation of in vitro generated α-syn oligomers and fibrils. **A)** Oligomers generated by resuspending lyophilised recombinant α-syn at high concentration (10mg/mL) incubated on ice and subsequently isolated using gel-filtration. Oligomers (O) are collected between 18–22 min. and monomers (M) between 38–43 min. Depicted gel filtration chromatogram is representative of oligomer elution profile observed in more than 10 oligomer preparations **B)** Representative graph of hydrodynamic radius in nm of isolated particles determined using DLS. Graph shows a merged image of the hydrodynamic radius (x-axis) of oligomers (dark grey) and monomers (white). Intensity of signal is depicted on the y-axis. (n = 3). **C)** Representative image of antigenicity of α-syn monomers, oligomers, and preformed fibrils to BD, FILA5 and MJF14 was determined using dot blot. Dots consist of 100ng protein spotted in duplicates (n = 3). Representative image of negative stain EM shows ultrastructure of native α-syn oligomer **D)** and **E)** ultrastructure of in vitro formed α-syn fibrils (n = 4).

Brief incubating of high concentration of α-syn at 0°C has previously been shown to spontaneously generate oligomeric species [19–21]. Using this method, in combination with size-exclusion chromatography, we successfully separated oligomers from monomers and obtained a pure oligomeric fraction (Fig 1A). Dynamic light scattering (DLS) confirmed the different sizes of the α-syn monomers and oligomers, showing two distinct species, with an average radius of 3.6 ± 0.3 and 49.7 ± 2.6 nm respectively (Fig 1B). Monomers, oligomers, and α-syn preformed fibrils all bind the α-syn specific antibody BD that recognize total α-syn levels, whereas oligomers and fibrils specifically binds the two aggregation-specific antibodies, FILA and MJF14 (Fig 1C) [10]. Transmission electron microscopy (TEM) revealed a twisted ribbon-like structure of the purified α-syn oligomers, which corresponds well with previous observation of oligomeric α-syn (Fig 1D) [22]. The α-syn oligomers clearly differ from the well-ordered structure and size of the preformed α-syn fibrils (Fig 1E).

### Stabilization of the α-syn oligomers using formaldehyde

**Fig 2:**
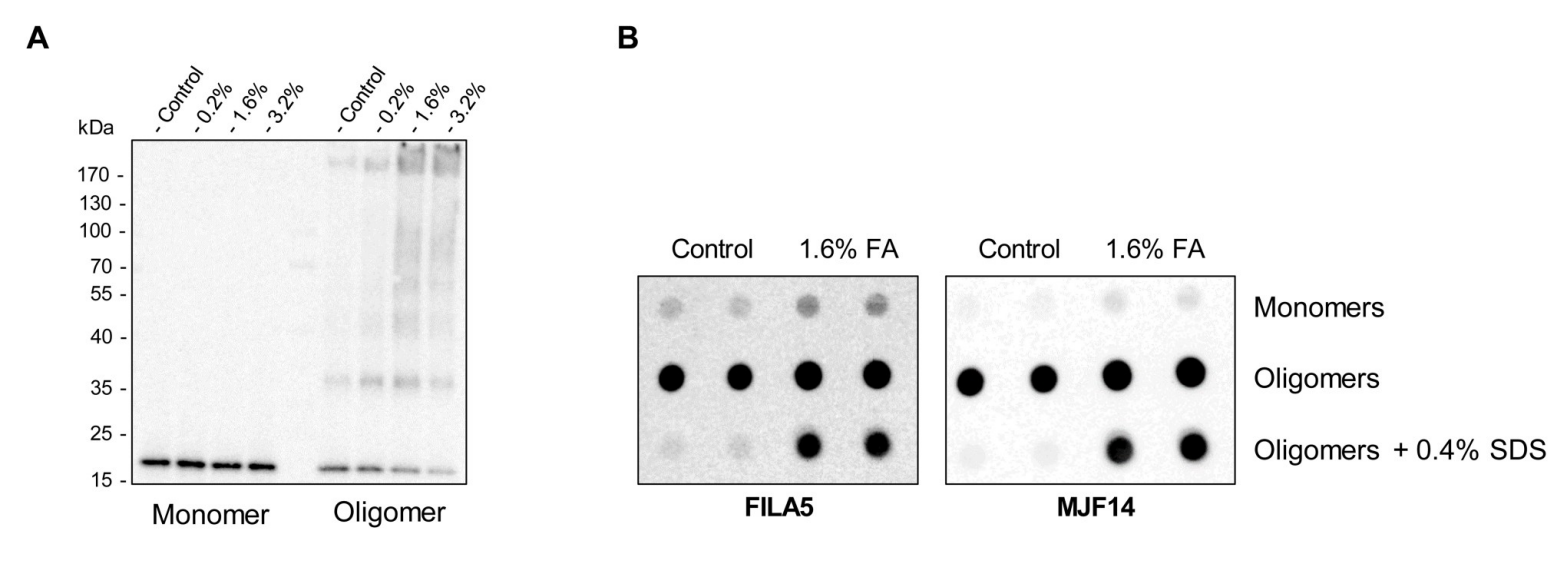
Cross-linking of α-syn monomers and oligomers. **A)** α-syn monomers and oligomers were cross-linked with FA at different concentrations for 30 min. Representative immunoblot of monomers (left) and oligomers (right) show ASY1 binding. Monomeric α-syn situates at ~17kDa. Depletion of the ~17kDa α-syn band and presence of ASY-1 reactivity in the stacking gel suggest efficient cross-linking upon FA treatment of oligomers. (n = 4) **B)** Representative dot blot of 100ng non-treated- or 1.6% FA cross-linked α-syn monomers and oligomers using aggregation-specific FILA5 and MJF14 antibody. Prior to dot-blot, one subset of native- and cross-linked oligomers were treated with 0.4% SDS for 1 h. to assess oligomer stability (n = 3).

α-syn oligomers consist of non-covalently bound monomers that dissociate into monomers upon boiling in SDS prior to SDS-PAGE (Fig 2A, control oligomer). However, incubation of the α-syn oligomers with increasing concentration of the small amine-reactive cross-linker, formaldehyde (FA), prior to SDS-PAGE, stabilized the α-syn multimers as evident from a depletion of the ~17kDa monomeric α-syn band (Fig 2A, 0.2%-3.6% oligomers). The retention of immunoreactivity in the stacking gel demonstrates the cross-linking of α-syn oligomers into large stable complexes (Fig 2A). By contrast α-syn monomers incubated with identical FA concentrations did not result in cross-linked-dependent depletion of the monomeric band nor accumulation of high molecular weight species (Fig 2A).

Visualization of FA-treated monomeric and oligomeric α-syn, using the aggregation-specific FILA and MJF14 antibodies, revealed that FA treated oligomers are also less prone to disassembly, as FILA and MJF14 antigenicity after 1h incubation with denaturizing SDS, is still observed (Fig 2B).

### Optimization of cross-linking

**Fig 3:**
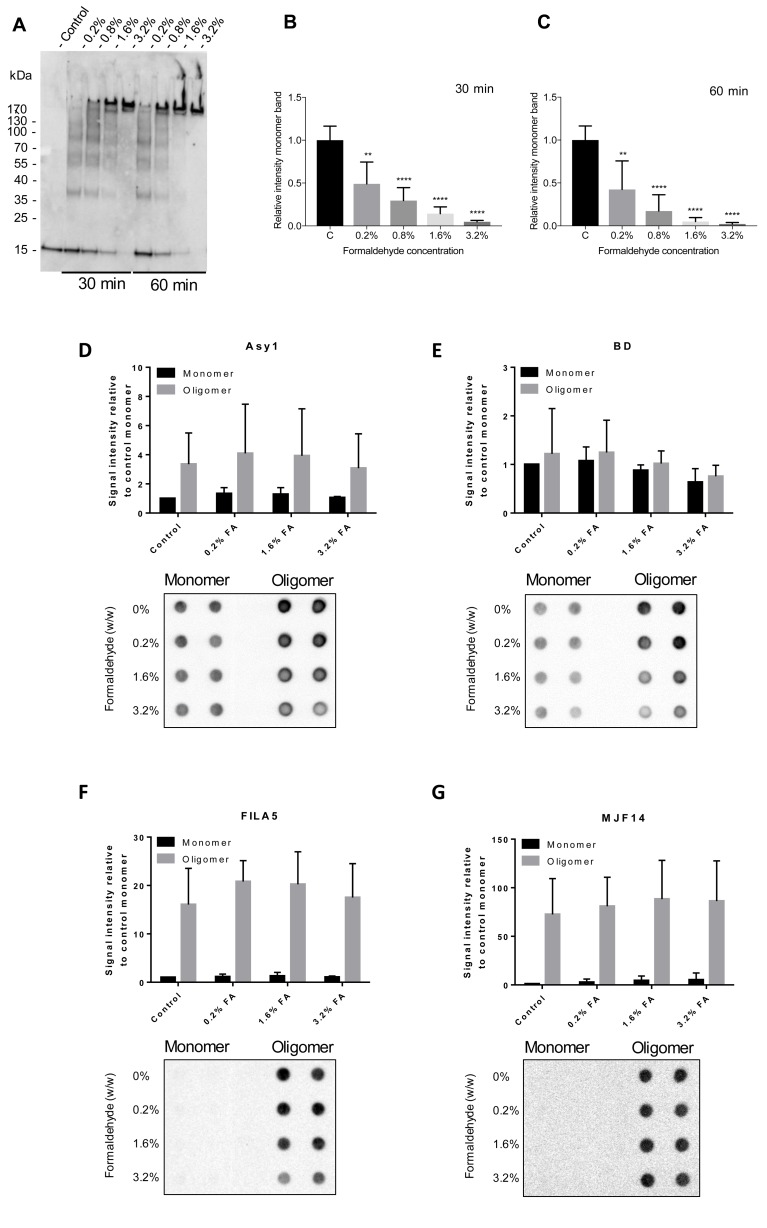
Optimization of α-syn oligomer cross-linking. **A-C)** α-syn oligomers were cross-linked with FA in a concentration of 0–3.2% for 30- or 60 min and subjected to immunoblot using ASY1 antibody to detect α-syn species (A). Monomer ~17kDa ASY-1 positive bands were quantified to assess degree of cross-linking and results are shown in B and C. Bar graphs show means ± SD obtained in three individual experiments. **D-G)** Monomers (black bars) and oligomers (grey bars) were cross-linked using different FA concentrations (0%-3.2% FA) for 60 min. The antigenicity of cross-linked monomers and oligomers were assessed via dot blot using ASY1 (D), BD (E), FILA5 (F) and MJF14 (G) antibodies. Figures show means ± SD of signal intensity, minus subtracted background signal, relative to non-cross-linked monomer samples (n = 4).

To avoid excessive and unnecessary chemical modification of the oligomers, we carried out titration experiments, aiming to find the lowest possible concentration of FA that efficiently stabilize α-syn oligomers. Using 0.2–3.2% FA and incubation for 30- or 60 min, we found that 1.6% and 3.2% FA after 60 min resulted in satisfactory cross-linking as measured by loss of the α-syn monomer-band (95 ± 4.6% and 98 ± 1.9% respectively), when resolved on SDS-PAGE (Fig 3A–3C). Further, we assessed if antigenicity of α-syn monomers or oligomers were affected by FA cross-linking. No difference was observed in oligomer recognition using antibodies BD, ASY1, FILA and MJF14 at any concentration of FA (Fig 3D–3G), although a trend of high FA concentrations masking the BD epitope of monomeric α-syn was observed (Fig 3E). 3.2% FA incubation generally results in a slightly higher cross-linking efficiency than 1.6% FA (Fig 3A and 3C), but we chose to continue with 1.6% FA for the remainder of the study for technical reasons. Primarily to avoid unnecessary dilution of the oligomers by adding larger volumes of Tris to quench the FA cross-linker.

### PFA stabilized α-syn retain biophysical and experimental properties

**Fig 4:**
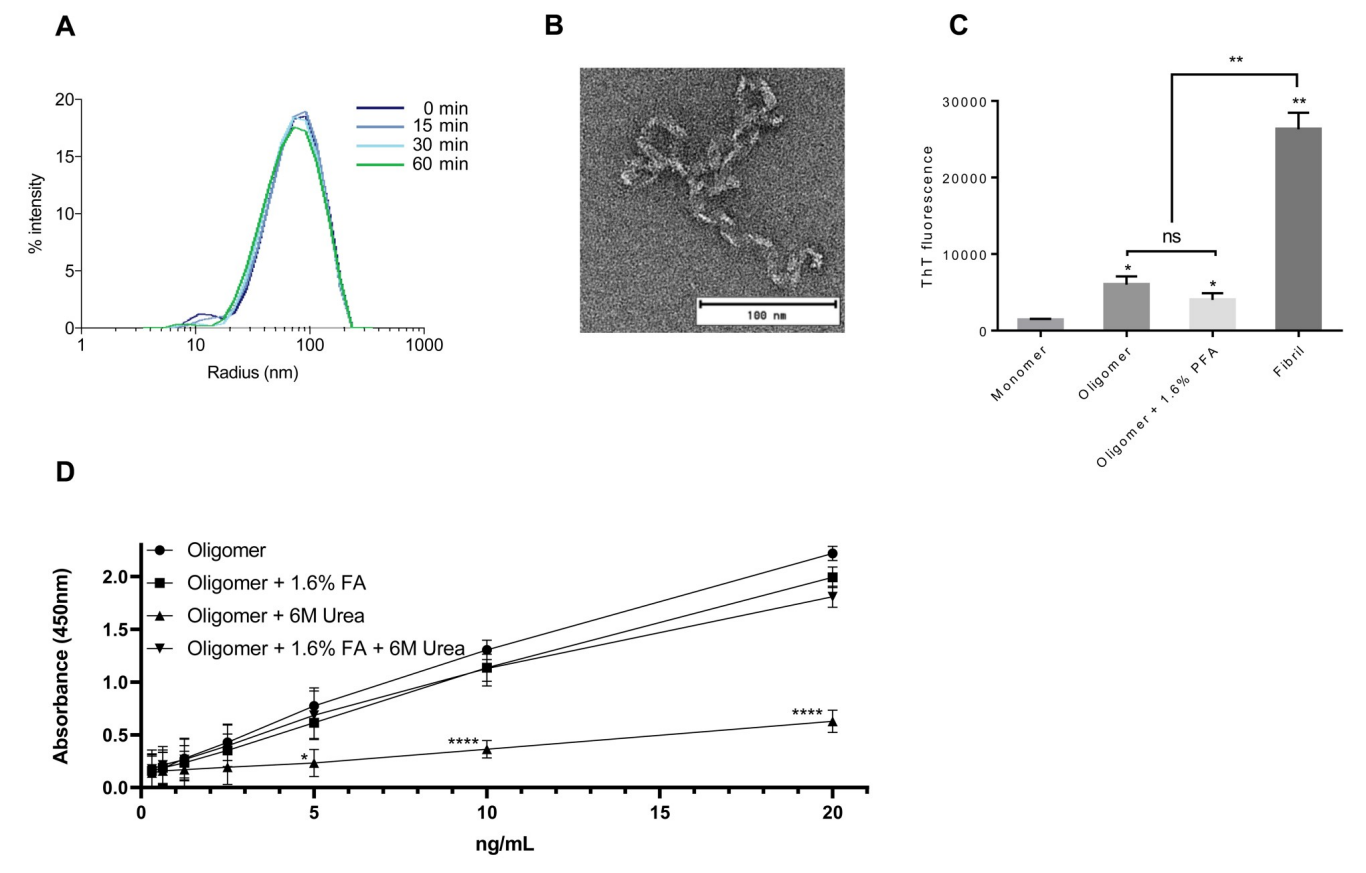
Oligomers cross-linked using FA maintain their size and structure and resist denaturation. **A)** Hydrodynamic radius of oligomers was monitored during 1.6% FA cross-linking using DLS. Measurements were taken before FA treatment (0 min) and 15-, 30- and 60 min after initiation of cross-linking (n = 3). **B)** Negative stain EM image shows ultrastructure of α-syn oligomer cross-linked for 60 min using 1.6% FA (n = 3). **C)** Thioflavin T fluorescence signal of 1.5μg α-syn monomer, oligomer, 1.6% FA cross-linked oligomer and preformed fibril (excitation at 450 nm and emission at 486 nm). Figure show means ± SD of three independent experiments. *p<0.05, **p<0.01, n.s. = not significant. **D)** Native and 1.6% FA cross-linked α-syn oligomer were treated with 6M Urea for 6h at RT or left untreated. Serial dilutions were analyzed by ELISA as previously described by Lassen et al. [23], utilizing the aggregated specific antibody MJF14. The levels are measured as absorbance at 450 nm. Two-way ANOVA for repeated measures followed by Tukey's multiple comparison test was used for ELISA experiment. Figure show means ± SD of three independent experiments. ****p<0.0001.

To test the biophysical properties of the 1.6% FA cross-linked oligomers, we performed a DLS time-course experiment, measuring the size-distribution of the oligomers after 0, 15, 30 and 60 min of 1.6% FA treatment. Throughout the experiment, the size of the oligomers was unchanged, suggesting that the oligomers are stabilized in their native structure and not cross-linked into larger complexes over time (Fig 4A). EM confirmed that the overall structure of the oligomers were kept after FA mediated cross-linking as these resembled native oligomers (Figs 1D and 4B). Furthermore, using Thioflavin T, we assessed the biophysical properties of our oligomers before and after cross-linking. Both cross-linked- and native oligomers exhibited enhanced β-sheet content relative to monomeric α-syn, yet far less compared to preformed α-syn fibrils (Fig 4C). This suggest that our oligomers are intermediates between monomeric- and fibrillary α-syn species, and that the nature of these oligomers are preserved upon cross-linking.

*In vitro* formed α-syn oligomers are useful in many experimental setups. One example, is the application of *in vitro* α-syn oligomers as internal standard in the newly developed aggregation-specific enzyme-linked immunosorbent assay (ELISA) based on the aggregation-specific MJF14 antibody [23]; an assay that effectively assess levels of α-syn oligomers in cell and animal models. Using this method, we compared the standard curves prepared from either native oligomers or FA cross-linked oligomers under different conditions (Fig 4D). We observed that ELISA standard curves using cross-linked oligomers were indistinguishable from those prepared with native oligomers (Fig 4D). Moreover, while pretreatment of native oligomers with 6M urea clearly abolish the MJF14 ELISA signal, FA cross-linked oligomers remained stable and created reliable standard curves despite exposure to high concentration urea (Fig 4D). Together, these findings demonstrate that FA treatment effectively cross-link and stabilize native oligomers without disturbing size, structure and antibody recognition of the oligomers.

## Discussion

Mounting evidence suggest a central role of soluble α-syn oligomers as toxic species relevant to the progressive spreading of α-syn pathology in the nervous system in synucleinopathies [24] and quantification of such species holds great potential as biomarkers [25–27].

In vitro formed α-syn oligomers represents a very heterogenous group that either presents as annular-, spherical-, circular-, rod shaped-, amorphous- and twisted ribbon-like structures dependent on the method of preparation and visualization [19, 21, 28]. Their diverse size, biochemical and structural properties likely make them dissimilar with respect to their presentation of antigens towards conformation specific-antibodies as demonstrated for insoluble α-syn strains [28, 29]. Furthermore, due to limited knowledge of the structure and size of *in vivo* oligomers we cannot say to which extend the different *in vitro* generated oligomer preparations resemble their *in vivo* counterparts. Therefore, neither of the different *in vitro* generated oligomer preparations can be claimed to truly represent oligomers found within the brain. Moreover the semistable nature of *in vitro generated* α-syn oligomers makes them unsuitable as analytical calibration standards in bioassays [20].

To overcome the limitations associated with oligomer instability, we have successfully cross-linked our preparation of *in vitro generated* α-syn oligomers using FA, thereby increasing stability towards SDS and urea denaturation. Our oligomer preparation binds neuronal proteins in a conformational specific manner [21, 22], and also share surface epitopes with patient-derived soluble- [10] as well as pathological insoluble α-syn [15] that are recognized by two conformational specific antibodies FILA and MJF14. The FA mediated cross-linking did not disturb the overall twisted ribbon-like oligomeric structure, the size of the oligomers, their antigenicity toward FILA and MJF14, the amount of amyloid structure nor the cross-linked oligomers applicability as calibrators in an α-syn oligomer specific ELISA assay [23]. It has yet to be experimentally determined whether this FA-mediated cross-linking protocol applies for all types of *in vitro* generated α-synuclein oligomers, but we propose that our FA cross-linked α-syn oligomer preparation could serve as calibrators that facilitates comparative and standardized biomarker studies in the synucleinopathies.

## Materials and methods

### Antibodies

Primary antibodies: α-syn monoclonal mouse-anti-α-syn (BD Biosciences, catalog no. 610787), polyclonal rabbit-anti-α-syn (ASY1 [30]), α-syn aggregate-specific antibodies FILA5 (polyclonal rabbit-antibody, in house generated [31]), and MJFR-14-6-4-2 (MJF14) (abcam, catalog no. 209538, rabbit monoclonal antibody, generated against full length α-syn protein filament). Secondary antibodies: horseradish peroxidase (HRP)-conjugated polyclonal rabbit-anti-mouse (Dako, P0260), HRP-conjugated polyclonal swine-anti-rabbit (Dako, P0217).

### Oligomer preparation

Production of α-syn and α-syn oligomers was performed as previously described [19, 21, 32]. In short, purified α-syn was dissolved in sterile PBS to a final concentration of 10mg/mL and incubated on ice for 30 minutes and vortexed once every 5 minutes during the incubation time. After incubation, α-syn was centrifuged at 12000g for 5 minutes and the supernatant loaded onto a Superdex™ 200 10/300 GL column (GE Healthcare). The column was eluted with PBS at a flow rate of 0.5 mL/minute. Oligomers were collected between 18–22 min and monomers after 38–43 min. All collected fractions were either directly used for further experimentation or snap frozen on dry ice and stored at -80°C.

### Fibril preparation

Lyophilized recombinant proteins were re-suspended in sterile PBS (Gibco) to a final concentration of 5mg/mL and placed in Eppendorf Thermotop shaker at 37°C, 1050 rpm for 5 days followed by centrifugation at 25.000g. Fibrils in pellet fraction was washed and re-suspended in PBS and adjusted to 2mg/mL.

### Cross-linking

Monomers and oligomers were cross-linked in various concentrations of formaldehyde at a final protein concentration of 10-15ng/μL depending on oligomer yield. Formaldehyde 37–38% w/w (PanReac AppliChem) was diluted in H_2_O and filtered (0,45μm) before use. Formaldehyde cross-linker was then added in a 2x concentration to monomer- or oligomer samples to obtain final concentration. Cross-linking was performed at 37°C and quenched with Tris pH 7.4 (two times molar concentration of FA) for 15 minutes at RT prior to dialysis in PBS using a pretreated Spectra/Por® 7 dialysis 10kD MWCO membrane (Spectrumlabs.com). Dialysis was performed ON at 4°C and PBS renewed 3 times during dialysis with a minimum of 4h intervals. Cross-linking for 1h using 1.6% final formaldehyde concentration was experimentally determined as most efficient.

### Immunoblotting

For Western blotting, samples were boiled for 5 min at 95°C for in loading buffer (50 mM Tris pH 6.8, 40% glycerol, 4% SDS, bromophenol blue) without reducing agent before loading onto a 4–16% PAGE gel (GenScript ExpressPlus™). Gels were blotted on PVDF membranes and fixed in 4% formaldehyde for 30 minutes and boiled in PBS for 5 minutes. For dot blotting 50-100ng of protein was blotted on nitrocellulose (Hydrobond-C Extra, GE Healthcare) and these filters were not fixed or boiled. All membranes were blocked in non-fat milk (TBS with 5% non-fat milk, 0.05% Tween 20 and 0.02% sodium azide) for 30 minutes at RT followed by incubation with primary antibodies overnight. membranes were washed in TBS-T (TBS with 0.1% Tween) and incubated with secondary antibodies for 1.5 hour. Membranes were washed, developed using ECL reagent (Pierce™, Thermo Scientific), and subsequently imaged on a LAS-3000 imaging system (Fuji). Blot quantification was done using ImageJ

### Size determination using dynamic light scatter

For size determination using Dynamic Light Scattering (DLS) samples were placed in a disposable cuvette and analyzed using a Wyatt Technology DynaPro NanoStar. Laser strength was set to 100% and scatter angle fixed at 90^o^. Total samples measurement where assembled via 10 consecutive 5 second measurements. Prior to analysis, samples were centrifuged for 3 minutes at >12000g. Data was analyzed with Dynamics V7.5.0.17.

### Negative staining transmission electron microscopy

Negative staining transmission electron microscopy (TEM) was performed as described previously [33, 34]. In brief, 3μL of sample was loaded onto a glow discharged carbon coated copper grid and stained with 2% uranyl formate. Images were collected using a Tecnai G2 Spirit TWIN electron microscope in combination with a Tietz TemCam-F416 CMOS camera at a magnification of 54000x to 67000x. Samples for EM were cross-linked in PBS and dialyzed, with three buffer changes, to TBS to avoid precipitation of phosphate with uranyl. Sample to dialyzing buffer volume ratio 1:100.

### Thioflavin T

To test for biophysical nature of α-syn species 1.5μg of α-syn monomer, oligomer, cross-linked oligomer and preformed fibril was tested for Thioflavin T signal. In brief, 100μl of 40μM Thioflavin T dissolved in measure buffer (90 mM glycine, pH 8,6) was added to 75μl of each sample and incubated for 5 min at RT before measuring Thioflavin T signal on an EnSpire 2300 Multilabel Plate Reader (PerkinElmer Life Sciences) (excitation at 450 nm and emission at 486 nm).

### Enzyme-linked immunosorbent assay

ELISA analysis was carried out as previously described [23]. In summary, 62.5 ng/mL MJF14 antibody and 0.5 μg/mL BD antibody were used as capture and detection antibody, respectively. 1,1 μg/mL native and cross-linked α-syn oligomer were incubated without or in the presence of 6 M urea for 6 hours at RT, before being analyzed by ELISA in at dilution ranging from 20 ng/mL to 313 pg/mL.

### Statistical analysis

Data was statistically analyzed and visualized using GraphPad Prism 7. Data was tested with a one-way ANOVA followed by Bonferroni post hoc analysis. For ELISA experiment, two-way ANOVA for repeated measures followed by Tukey's multiple comparison test was used. Differences were considered significant for *p<0.05, **p<0.01, ***p<0.001, ****p<0.0001. Data is presented as means ± SD.
